# Bovine Milk–Derived Extracellular Vesicles as Emerging Drug Delivery Platforms: Current Advances, Applications and Challenges

**DOI:** 10.1002/jex2.70160

**Published:** 2026-06-27

**Authors:** Martina Brattini, Michelle D'Urso, Sofia Mariotto, Elena Butturini

**Affiliations:** ^1^ Department of Neuroscience, Biomedicine and Movement Science Section of Biological Chemistry University of Verona Verona Italy

**Keywords:** drug delivery, extracellular vesicles, milk

## Abstract

Extracellular vesicles (EVs) are nanosized lipid bilayer particles naturally secreted by cells, mediating intercellular communication and transporting diverse bioactive molecules. Among alternative EV sources, bovine milk‐derived EVs (BMEVs) have emerged as promising platforms for drug delivery due to their accessibility, scalability, stability and promising biocompatibility, although their long‐term safety profile, particularly for engineered or cargo‐loaded formulations, still requires further investigation. BMEVs can encapsulate small molecules, natural polyphenols and nucleic acids, enhancing bioavailability, protecting cargo from degradation and facilitating functional delivery. Recent studies demonstrate their potential for oral administration and their intrinsic therapeutic properties, including antioxidant, anti‐inflammatory, and immunomodulatory activities. Moreover, the increasing number of patents highlights their translational and commercial relevance in therapeutic, cosmetic and nutraceutical applications. Despite these promising features, challenges remain in standardizing isolation protocols, optimizing cargo loading, ensuring batch reproducibility and evaluating long‐term safety. Addressing these gaps is essential to enable clinical translation and establish BMEVs as versatile drug delivery platforms.

**Trial Registration**: ClinicalTrials.gov identifier: NCT07402083

## Graphical abstract

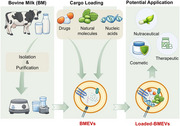

## 1 Introduction

Extracellular vesicles (EVs) are lipid bilayer‐enclosed particles naturally released by cells that cannot replicate on their own (Welsh et al. 2024). They are produced by almost all cell types and constitute a heterogeneous population that can be classified according to their biogenesis, size and release mechanism (El Andaloussi et al. 2013). The two main subtypes include exosomes, small vesicles (40–120 nm) released upon the fusion of multivesicular bodies with the plasma membrane, and microvesicles, larger vesicles (up to 1000 nm) formed by the direct outward budding of the plasma membrane. Additional vesicle populations include microsomes and apoptotic bodies (1000–4000 nm). Because no unique molecular markers reliably distinguish EV subtypes and current isolation approaches do not allow selective purification of individual populations, the International Society of Extracellular Vesicles (ISEV) introduces the Minimal ISEV (MISEV) guidelines and recommends the use of operational terminology based on physical or biochemical characteristics rather than subtype‐specific definitions (Welsh et al. 2024; Du et al. 2023). They provide standards for EV nomenclature, isolation and characterization, and emphasize functional validation and rigorous controls to ensure reproducibility and transparency in EV research. A summary of the MISEV‐recommended characterization criteria is provided in Table 1.

**TABLE 1:** Overview of MISEV reccomandationfo EV characterization. NTA: nanotracking analysis; TRPS: Tunable Resistive Pulse Sensing; TEM: Transmission Elecron Microscopy.

Category	Recommendation	Methods	Suggested values/markers
Physical characterization	Assess particle concentration and size distribution	NTA TRPS Flow cytometry	∼30–200 nm (small EVs), up to ∼1 µm (large EVs)
Morphology	Confirm vesicular structure	TEM Cryo‐EM	Cup‐shaped lipid bilayer vesicles
Positive markers	Confirm EV enrichment	Western blot Flow cytometry	CD9, CD63, CD81, TSG101, ALIX, HSP70
Negative markers	Exclude contaminants	Western blot Flow cytometry	Calnexin, GM130, cytochrome c, albumin, apolipoproteins

Originally described as cellular waste disposal structures, EVs are now recognized as a central mediator of intercellular communication in numerous physiological and pathological processes (Wiklander et al. 2019; Kimiz‐Gebologlu and Oncel 2022). This functional role has driven increasing interest in their application as therapeutic agents, biomarkers, and, more recently, as drug delivery systems. Compared with synthetic nanocarriers, EVs have several advantageous properties. They present intrinsic biocompatibility and low immunogenicity, protect their cargo from degradation, and are able to cross biological barriers like the blood‐brain barrier (Kimiz‐Gebologlu and Oncel 2022; van der Koog et al. 2022). These features have prompted the development of engineered or cargo‐loaded EVs capable of delivering proteins, nucleic acids and small molecules to recipient cells with high efficiency (Wang and Luo 2022; Bettin et al. 2024; Evers et al. 2022; Fuhrmann et al. 2015; Chen, Sun, et al. 2021; Chen, Chen, et al. 2021).

EVs can be isolated from various biological fluids and cell culture conditioned media (Du et al. 2023; Bano et al. 2021). Despite their considerable potential, EV‐based delivery strategies face important limitations, especially low yield, poor reproducibility of cargo loading and challenges associated with scalable production (Kimiz‐Gebologlu and Oncel 2022; Mondal et al. 2023). Many of these limitations are intrinsically linked to the widespread use of cell‐conditioned media as the primary source of EVs in the majority of studies investigating their application. To overcome these issues, increasing attention has been directed toward alternative natural sources of EVs, such as fruit and vegetable juices (Perut et al. 2021; Long et al. 2023; Bokka et al. 2020; Dad et al. 2021; Steć et al. 2025), as well as milk from different animal species (Saeed et al. 2024; Wijenayake et al. 2021; Samuel et al. 2023). Notably, these sources are easily accessible, cost‐effective, biocompatible and potentially suitable for large‐scale production, while also offering opportunities for the valorization of industrial by‐products and thus improving the sustainability of EV manufacturing (Langellotto et al. 2025; Giancaterino and Boi 2023).

Among the alternative sources investigated to date, bovine milk is one of the most extensively studied. Milk represents a particularly rich source of EVs released by mammary epithelial, immune, stem and adipocyte cells present in the mammary glands, providing the opportunity to obtain high amounts of EVs (Admyre et al. 2007; Di Fabrizio et al. 2026; Prabhu et al. 2026). Accumulating evidence indicates that milk‐derived EVs are involved in molecular communication between mother and infant, and can also mediate cross‐species molecular transfer, highlighting their capacity to deliver functional biomolecules to recipient cells (Prasadani et al. 2024; Barathan et al. 2024). Moreover, the widespread consumption of bovine milk across age groups suggests a potentially favourable biocompatibility profile of its vesicular components. However, the safety of isolated, concentrated, engineered or cargo‐loaded BMEVs cannot be directly assumed from dietary milk consumption and still requires systematic evaluation. Consequently, growing interest has emerged in the use of bovine milk‐derived EVs (BMEVs) as a promising platform for drug delivery applications (Sanwlani et al. 2020).

Despite their promising properties, BMEVs have not yet entered clinical testing, and their future development will require the establishment of standardized production protocols and a clear regulatory pathway for EV‑based therapeutics.

This review summarizes recent advances in the use of BMEVs as a drug delivery tool, highlighting their key advantages and providing representative examples of therapeutic cargo delivery. In addition, current applications of BMEVs in commercial products and patents are discussed. Finally, the critical aspects hampering their clinical translation are highlighted.

## 2 Advantages of Milk‐Derived EVs as Drug Delivery Vehicles

**FIGURE 1:**
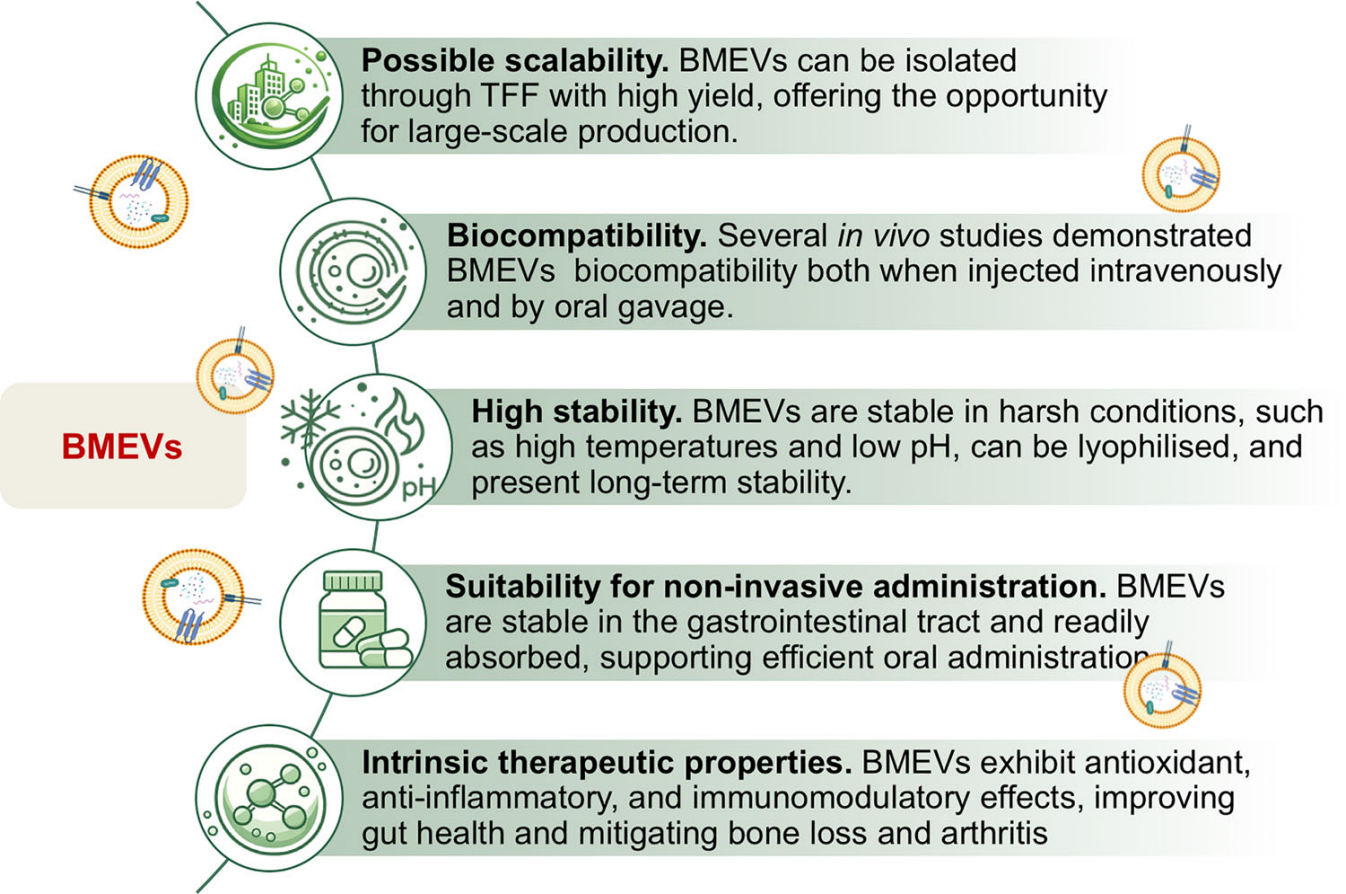
Advantages of BMEVs as a drug delivery vehicle. BMEVs present some characteristics making them a promising candidate to be used as a drug delivery vehicle.

In recent years, BMEVs have attracted increasing interest as drug delivery vehicles. Milk represents a unique biological source that offers several advantages, which can be summarized into five main aspects (Figure 1).

### 2.1 Scalable Isolation

Most of the current studies rely on cell‐conditioned media (CCM) for EVs isolation; however, these methods require large culture volumes and involve protocols that are difficult to scale (Kimiz‐Gebologlu and Oncel 2022). In contrast, milk offers a highly efficient alternative: large amounts of EVs can be isolated from relatively small volumes, a critical advantage for translational drug delivery applications (Marsh et al. 2025; Munir et al. 2023). This makes milk not only a more scalable source but also a practical and cost‐effective option for producing high EV concentrations.

For example, Mukhopadhya et al. reported significantly lower particle concentration in mesenchymal stem CCM than in milk; however, comparable efficacy in doxorubicin encapsulation and delivery was observed (Mukhopadhya et al. 2023). Similarly, Onizuka et al. reported an approximately two‐fold higher particle concentration in milk‐derived EVs than in EVs isolated from the MDA‐MB‐231 human breast cancer cell line conditioned medium (Onizuka et al. 2023). Together, these findings support the feasibility of large‐scale EV production from milk.

BMEVs can be isolated using standard EV purification techniques based on their physical and chemical properties (Table 2). Differential ultracentrifugation separates EVs from other contaminants according to sedimentation rate, whereas density gradient ultracentrifugation separates particles based on buoyant density. In contrast, size‐exclusion chromatography and ultrafiltration rely on particle size and molecular weight. In addition, affinity‐based approaches, such as immune‐magnetic beads, allow the recovery of specific EV sub‐populations, although their limited scalability and cost may limit broader application.

**TABLE 2:** Overview of methods mainly used for the isolation and purification of EVs.

Methods	Separation principle	Advantages	Disadvantages
Differential ultracentrifugation	Sedimentation rate	Simple to perform High sample volume Minimal risk of contamination	Can co‐isolate contaminants Time‐consuming Expensive equipment Poor scalability
Density‐gradient ultracentrifugation	Buoyant density	Higher purity	Labour‐intensive Low scalability Expensive
Size‐exclusion chromatography	Particle size	No high pressure or shear forces applied to samples Separate different EVs subpopulation Low cost Possible automatization	Limited resolution for similar sizes Limited volume of the initial Sample High dilution of final EVs
Ultrafiltration	Particle size	Fast Scalable	Risk of vesicle deformation Membrane fouling
Immune‐magnetic beads	Specific molecular binding	Isolates specific EV sub‐populations	Expensive Low scalability
Tangential flow filtration	Membrane cut‐off & tangential flow	Scalable Preserves EV integrity Minimizes fouling	Requires specialized equipment Needs sample optimization

Among available isolation strategies, tangential flow filtration (TFF) has emerged as a particularly promising approach for large‐scale BMEVs production (Giancaterino and Boi 2023). By employing membranes with defined molecular cut‐offs and a tangential flow configuration that minimizes membrane fouling, this method enables the processing of large volumes and preserves vesicle integrity and biological activity (Giancaterino and Boi 2023; Rhim et al. 2023; Marsh et al. 2021). Consistently, several studies have successfully applied this technique to isolate milk EVs and supported their suitability for translational and commercial applications (del Saz‐Lara et al. 2025; Schifano et al. 2025).

**FIGURE 2:**
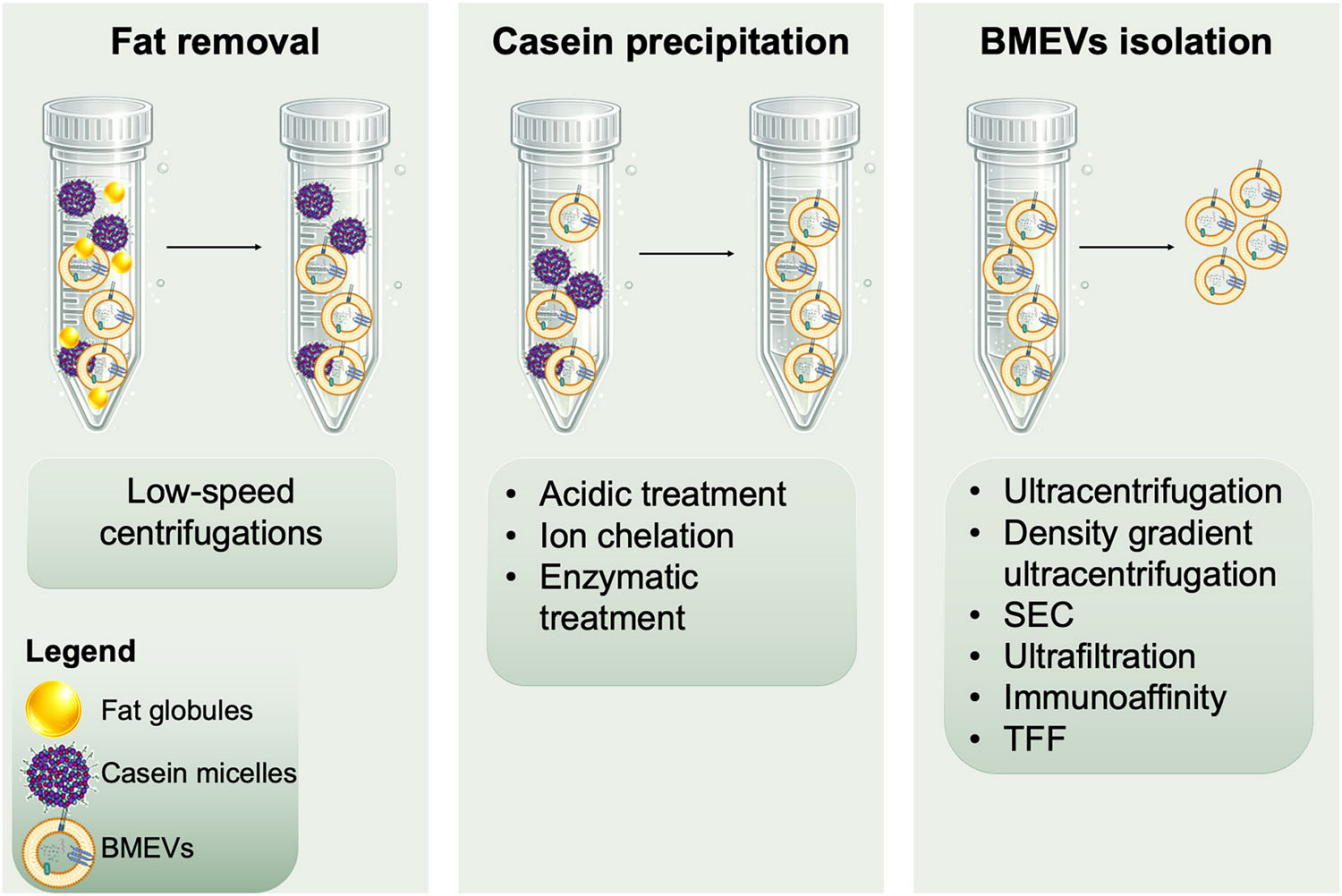
Schematic overview of BMEV's isolation. Due to the complex composition of milk, preliminary steps are required to remove fat globules and casein micelles, typically through low‐speed centrifugation followed by acid precipitation, ion chelation or enzymatic treatment. After these pre‐clearing steps, BMEVs can be isolated using different methods depending on the intended downstream application.

Because milk is a complex matrix rich in fat globules and casein micelles, additional purification steps are typically required to improve EV purity (Figure 2) (Salehi et al. 2024). Preliminary centrifugation steps remove fat globules, whereas casein micelles can be eliminated through acid precipitation, calcium chelation (e.g., EDTA) or enzymatic coagulation with chymosin (Cetinkaya et al. 2024; Wang et al. 2024). Specifically, the addition of acetic acid or hydrochloric acid lowers the pH to the isoelectric point of casein, causing its aggregation and precipitation (Rahman et al. 2019). Alternatively, EDTA chelates calcium ions destabilizing casein micelles and promoting their precipitation (Weiskirchen et al. 2023). Finally, chymosin specifically cleaves κ‐casein, destabilizing the micelles and inducing casein precipitation, which facilitates subsequent efficient EV purification from milk (Liu et al. 2021; Kethireddipalli and Hill 2015). Each strategy presents advantages and limitations. Rahman et al. demonstrated that both acidification and EDTA treatment allow the efficient removal of casein micelles from different kinds of milks; however, in accordance with del Saz‐Lara et al., acid addition decreased particle concentrations and degraded some EVs protein markers, showing the aggressive nature of this treatment (del Saz‐Lara et al. 2025; Rahman et al. 2019). At the same time, Mendel‐Martinez et al. and Cetikaya et al., observed higher EVs concentrations and EVs marker enrichment when chymosin was used; however, this procedure requires more time and an additional step of centrifugation with respect to other treatments (Cetinkaya et al. 2024; Medel‐Martinez et al. 2024).

Although no consensus protocol has yet been established, most studies agree that dedicated casein‐removal steps are essential, since ultracentrifugation alone is insufficient to eliminate casein contamination.

### 2.2 Biocompatibility and Safety Profile

Biocompatibility is a key requirement for any delivery platform. While synthetic nanoparticles such as liposomes are widely used clinically, they can exhibit rapid clearance, trigger immune responses upon repeated administration, and accumulate in clearance organs (van der Koog et al. 2022). In contrast, the natural origin of BMEVs suggests an intrinsically favourable safety profile.
Given that bovine milk is widely consumed and generally well tolerated, BMEVs are considered potentially low‐immunogenic and safe for biomedical applications. This assumption is supported by various in vivo studies. Somiya et al. reported no systemic toxicity or increased concentrations of inflammatory cytokines after either single or repeated intravenous administration of BMEVs in mice over 14‐day period, even at higher doses and any anaphylaxis reactions after five weekly intravenous injections (Somiya et al. 2018). Similarly, Kumar et al. demonstrated biocompatibility of BMEVs after oral administration in rats, as evidenced by normal haematological and biochemical parameters and absence of histopathological alteration in major organs (Kumar et al. 2025). Moreover, Munagala et al. observed that BMEVs orally administered to rats were broadly distributed across the tissues, whereas intravenously injected vesicles accumulated mainly in the liver. Importantly, no systemic toxicity was detected in either case following short‐ or prolonged‐exposure (Munagala et al. 2016).
Additional in vivo evidence supports the tolerability of milk‐derived vesicles as delivery platforms. Matsuda et al. evaluated the safety of bovine milk‐derived nanovesicles, including vesicles modified for RNA delivery, in zebrafish and mice, with no detectable systemic toxicity, developmental defects or inflammatory responses following repeated administration (Matsuda et al. 2020). More recently, studies exploring the biological activity of BMEVs in vivo have confirmed preserved tissue integrity and absence of adverse effects, even when vesicles were administered at relatively high concentrations (Ribas‐Maynou et al. 2025). Collectively, these findings reinforce the favourable acute tolerability of BMEVs; however, it is important to note that all available in vivo studies have evaluated only short‑term or sub‑chronic exposure, and no work has yet examined long‑term biodistribution, immune modulation or potential accumulation. These gaps highlight the need for systematic chronic‑toxicity studies to fully establish the long‑term safety profile of BMEVs.

### 2.3 High Storage Stability

Stability during storage and transport is a critical parameter for effective delivery systems. Freshly isolated milk EVs are commonly stored at –20°C or –80°C for long‐term preservation, and at 4°C for short‐term storage. These conditions generally preserve vesicle size, although some studies report a gradual reduction in particle concentration over time (Munagala et al. 2016; Wu et al. 2025). Wu et al. reported that curcumin‐loaded BMEVs remain stable in size for up to 7 days at 4°C and for up to 1month at –80°C, with a minor effect of temperature up to 80°C on curcumin stability compared to free curcumin (Wu et al. 2025). Similarly, Munagala et al. observed no significant changes in vesicle sizes and biological activity of milk derived EVs at –80°C for up to 18 months (Munagala et al. 2016).

The recent COVID‐19 pandemic crisis highlighted the logistical and economic challenges associated with cold‐chain distribution, prompting increased interest in room temperature‐stable formulations. Lyophilization represents an attractive preservation strategy, as it enables long‐term storage by removing water from frozen samples under vacuum. Different studies have demonstrated that milk EVs can be lyophilized while preserving structural and functional integrity, although the process may induce mechanical stress or pH shifts (Lu et al. 2024; Charoenviriyakul et al. 2018). The addition of cryoprotectants and bulking agents can mitigate these effects. For example, BMEVs formulations containing trehalose and tryptophan preserved vesicle concentration, size and biological activity for up to 6 months at room temperature (Dogan et al. 2025), whereas the trehalose–mannitol mixture maintained stability for months even at elevated temperatures (Lu et al. 2024). Overall, the stability of extracellular vesicles following lyophilization appears to depend on multiple factors, including the cellular source that reflect differences in vesicle lipid and protein composition as well as the formulation and presence of cryoprotectants; nevertheless, lyophilization remains a promising strategy for the long‐term preservation and distribution of EV‐based products that must be optimized for each EV source (Bosch et al. 2016; Susa et al. 2023; Frank et al. 2018).

### 2.4 Suitability for Non‐Invasive Administration

Beyond storage stability, the route of administration is a critical consideration in drug delivery. Oral administration provides a non‐invasive and cost‐effective route, as well as avoiding the sterility requirements associated with injections. Nevertheless, many therapeutic agents show poor oral bioavailability due to degradation within the gastrointestinal tract. In this context, milk‐derived EVs have emerged as promising candidates for oral delivery, as industrial processing may select a resilient subset of vesicles capable of withstanding harsh conditions, potentially supporting their use as oral delivery systems, although the extent of vesicle integrity and functional uptake after gastrointestinal transit remains incompletely understood (Colella et al. 2024; Kleinjan et al. 2021).

Several studies suggest that BMEVs may cross the intestinal barrier under specific experimental conditions, although whether they remain fully intact throughout the gastrointestinal tract remains controversial (Schifano et al. 2025; Roerig et al. 2021; Tong et al. 2023). Tong et al. observed that labelled BMEVs were detected in the intestine and the colon after 1 and 6 h after oral administration, respectively, and alleviated intestinal inflammation in a murine model of ulcerative colitis (Tong et al. 2023). The molecular mechanism is related to the inhibition of TLR4‐NF‐κB and NLRP3 signalling that restores Treg and Th17 cells balance and the gut microbiota (Tong et al. 2021, 2023). Encapsulation studies further indicate that BMEVs can protect therapeutic cargo, such as insulin, from enzymatic degradation in simulated gastric and intestinal fluids (Wu et al. 2022).

Conversely, other studies report conflicting results regarding BMEV stability under digestive conditions. Using an in vitro infant gastrointestinal digestion model, exposure of BMEVs to digestive enzyme but not bile salts, resulted in reduced particle concentration, altered morphology and decreased expression of EV markers such as CD9 and TSG101 (Oliver et al. 2024). These findings suggest that the primary cause of BMEV's disruption is the activity of digestive enzymes. Similarly, Seegobin et al. found that the EV lipid bilayer was damaged after exposure to highly acidic conditions and to gastric and intestinal fluids. Furthermore, in vivo administration of BMEVs in a colitis mouse model showed no beneficial therapeutic effects, regardless of whether the treatment was given orally or rectally (Seegobin et al. 2025). Interestingly, Xu et al. found that sublingual administration of liraglutide‐loaded BMEVs resulted in higher bioavailability and improved therapeutic efficacy compared to oral gavage administration (Xu et al. 2022).

The discrepancies observed across studies may arise from several methodological and biological factors. Differences in EV isolation and purification protocols can influence vesicle composition, purity and stability. In addition, variability in storage conditions and industrial processing may affect membrane properties and resistance to gastrointestinal stress. Another important aspect is the use of different experimental models, since in vitro digestion systems do not fully reproduce the complexity of the in vivo gastrointestinal environment. Moreover, studies often evaluate different endpoints, including vesicle integrity, intestinal uptake, cargo protection or therapeutic efficacy, which may lead to apparently conflicting conclusions. Importantly, partial disruption of the EV membrane during digestion may not necessarily correspond to a complete loss of biological activity, as some bioactive molecules may still remain functional. Differences in dosage, administration route and disease model may further contribute to the heterogeneous outcomes reported in the literature.

Overall, although BMEVs show promising features for oral delivery applications, current evidence regarding gastrointestinal stability, intact vesicle uptake, cargo preservation and therapeutic efficacy remains heterogeneous, highlighting the need for further standardized and mechanistic investigations.

## 3 Intrinsic Biological Properties

BMEVs possess intrinsic bioactivities due to their endogenous cargo of lipids, proteins and nucleic acids (Prasadani et al. 2024; Jabłońska et al. 2024). Importantly, these intrinsic biological effects of native BMEVs should be clearly distinguished from the delivery‐related effects observed in engineered or cargo‐loaded BMEVs. While native vesicles exert endogenous regulatory activities through their naturally occurring cargo, loaded BMEVs are primarily investigated as delivery platforms for exogenous therapeutic agents, involving distinct mechanistic and translational considerations. Through their natural function in intercellular communication, these vesicles can transfer bioactive molecules to recipient cells and modulate cellular signalling pathways (Li et al. 2026). A growing body of evidence indicates that BMEVs act as natural antioxidants. Intrinsically, BMEVs transport antioxidant enzymes such as superoxide dismutase (SOD), catalase and glutathione peroxidase (GPX), which contribute to the neutralization of reactive oxygen species (ROS) and protect cells from oxidative damage (Wang et al. 2021, 2025; Xiang et al. 2023). In addition, membrane‐associated proteins may further contribute to antioxidant protection by scavenging free radicals, inhibiting lipid peroxidation and preventing iron‐catalyzed ROS generation through modulation of iron homeostasis and metal‐binding processes.

Beyond their intrinsic antioxidant cargo, BMEVs can also modulate the antioxidant response of recipient cells. Wang et al. showed that pretreatment with BMEVs protected the intestinal crypt epithelial cells IEC‐6 from H_2_O_2_‐induced oxidative stress by promoting cell proliferation, decreasing intracellular levels of ROS and malondialdehyde (MDA), and increasing the activity of antioxidant enzymes as SOD and GPx (Wang et al. 2021). Consistently, Liang et al. reported that BMEVs reduced oxidative stress and ferroptosis in *Klebsiella pneumoniae*‐infected bovine mammary epithelial cells in vitro and in murine mammary tissues in vivo (Liang et al. 2025). This regulatory effect may be mediated by EV‐associated miRNAs, miR‐146a and miR‐155, which activate the Nrf2 signalling pathway and promote the transcription of antioxidant genes including HO‐1, GPX and SOD2 (Chen, Sun, et al. 2021; Chen, Chen, et al. 2021).

BMEVs also display anti‐inflammatory and immunomodulatory properties in several in vitro and in vivo inflammation‐related disease models. Evidence for these effects is particularly strong in the gastrointestinal tract. Mecocci, Trabalza‐Marinucci, et al. (2022) demonstrated that BMEVs treatment reduced pro‐inflammatory cytokine production and partially restored epithelial homeostasis in an in vitro co‐culture model of intestinal inflammation (Mecocci, Ottaviani, et al. 2022). Similarly, studies in LPS‐stimulated RAW264.7 macrophages have shown that BMEVs attenuate inflammatory responses by modulating the NF‐κB pathway and reducing pro‐inflammatory cytokines production (Cheng et al. 2025).

Consistent with these findings, several studies have reported that BMEVs alleviate intestinal inflammation and improve intestinal barrier integrity by modulating key signalling pathways, like TLR4‐NF‐ƙB axis and NLRP3 inflammasome activation. These effects are accompanied by increased mucin production and up‐regulation of tight junction proteins, ZO‐1 and Occludin (Tong et al. 2021).

In vivo evidence further supports the protective role of BMEVs in intestinal inflammatory disorders. In a murine model of colitis, BMEVs administration alleviated disease symptoms and promoted intestinal epithelial regeneration, suggesting their potential therapeutic application in inflammatory bowel disease (Reif et al. 2020). Similarly, Li et al. demonstrated that BMEVs mitigated intestinal injury in both in vitro and in vivo models of necrotizing enterocolitis by preventing disease progression and increasing goblet cell abundance and mucin production (Li et al. 2019).

In addition, BMEVs may also modulate intestinal immunity through interactions with the gut microbiota. Oral administration of BMEVs in mice altered the profile of gut microbial metabolites and increased the expression of genes involved in intestinal barrier function and immune regulation (Tong et al. 2020).

Interestingly, the anti‐inflammatory and immunomodulatory properties of BMEVs are not limited to the gastrointestinal system. Pieters et al. reported that local administration of BMEVs to cartilage explants from osteoarthritis patients reduced glycosaminoglycan release and downregulated cartilage‐degrading enzymes in human chondrocytes. These protective effects were attributed to bioactive components TGF‐β and miR‐148a contained within BMEVs, which regulate chondrocyte homeostasis and cartilage integrity (Pieters et al. 2022). Moreover, Oliveira et al. demonstrated that BMEV treatment improved bone microarchitecture and reduced osteoclast activity in mildly obese and ovariectomized mouse models, effects linked to modulation of the RANKL/OPG signalling axis involved in bone remodelling (Oliveira et al. 2020).

Importantly, the biological activity of BMEVs is also influenced by the lactation stage, reflecting the dynamic evolution of milk composition along the lactation curve. EVs derived from colostrum are typically enriched in immune‐related proteins, growth factors and regulatory miRNAs, and have been associated with enhanced immunomodulatory activity, and protective effects, particularly in the context of epithelial damage and early‐life immune system priming. By contrast, EVs from mature milk appear to be more involved in the maintenance of epithelial barrier function and systemic homeostasis, displaying a more balanced regulatory profile with sustained but less pronounced immune‐activating properties (Liu et al. 2024; Santoro et al. 2023). These observations support the notion that lactation timing represents a relevant biological variable contributing to functional heterogeneity of BMEVs.

Collectively, these findings highlight the broad intrinsic therapeutic potential of BMEVs across multiple biological systems. Nevertheless, further studies are required to clarify their mechanisms of action and to determine how parameters such as purity, dose and treatment duration influence their efficacy.

## 4 Examples of Delivery Applications

**FIGURE 3:**
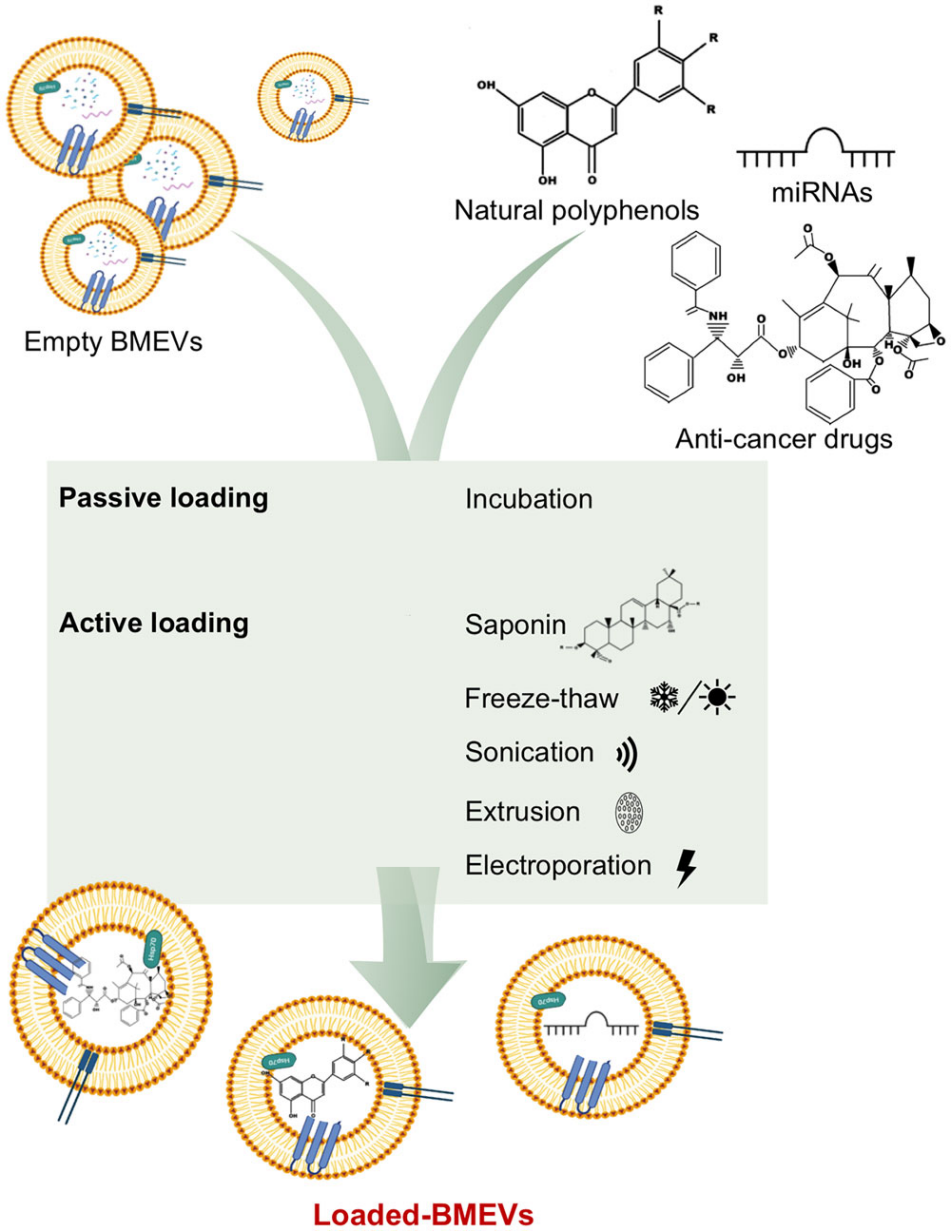
Overview of the main methods for loading therapeutic cargo into BMEVs. Various molecules, including natural polyphenols, anticancer drugs and miRNAs, have been successfully encapsulated. Loading approaches are generally classified as passive or active. Passive loading relies on simple incubation of the compound with BMEVs, whereas active methods transiently disrupt vesicle membranes using techniques such as electroporation, sonication, extrusion, freeze–thaw cycles or saponin treatment.

Over the years, BMEVs have been extensively investigated as drug delivery vehicles for various compounds. To this end, a range of loading strategies has been used, which can be broadly classified as passive and active (Figure 3). Passive loading methods are based on the simple incubation of BMEVs with the compound of interest and are most suitable for hydrophobic molecules that can interact with the vesicle lipid bilayer. This approach is easy, cost‐effective, and does not require specialized equipment. However, it typically exhibits low encapsulation and loading efficiency and requires optimization of incubation conditions (Jiang et al. 2024).

Active loading strategies enable compound incorporation through transient disruption of the EVs surface using mechanical (electroporation, sonication, extrusion and freeze–thaw cycles) and chemical (detergents) techniques. Each method presents specific advantages and limitations, and the optimal choice depends on the physicochemical properties of the cargo and the intended application. Although passive methods may exhibit lower loading efficiency, the active approaches can alter vesicle surfaces and lead to partial loss of their intrinsic cargo, which may itself exert beneficial effects (Marsh et al. 2025; Jiang et al. 2024).

This section summarizes representative examples of BMEVs used as drug delivery vehicles of small molecules, natural polyphenols and nucleic acids.

### 4.1 Small Molecules

BMEVs have been used to improve the bioavailability and reduce the toxicity of anticancer drugs. One example is paclitaxel, a first‐line chemotherapeutic agent for multiple cancers. It has been observed that paclitaxel exhibits higher antitumour efficacy when loaded into BMEVs and administered orally in a lung tumour xenograft mouse model, compared with the intravenously administered free drug. Importantly, paclitaxel‐loaded BMEVs did not induce significant systemic or tissue toxicity, potentially overcoming the adverse effects associated with intravenous injections of free drug (Agrawal et al. 2017).

Similarly, dihydroartemisinin (DHA), an antimalarial drug with recognized anticancer properties, induced stronger cytotoxicity and apoptosis in B16F10 melanoma cells when loaded into BMEVs compared to its free form. In vivo, orally administered DHA‐loaded BMEVs increased bioavailability, improved therapeutic efficacy and reduced metastasis formation in melanoma‐bearing Swiss mouse model (Kumar et al. 2025).

More recently, BMEVs have also been investigated as delivery vehicles for sorafenib in hepatocellular carcinoma. In this study, sorafenib‐loaded BMEVs exhibited enhanced cellular uptake, improved antitumour activity, and lower cytotoxicity toward normal cells compared to the free drug, further supporting the potential of BMEVs as scalable and biocompatible nanocarriers for anticancer therapy (Salehi et al. 2026).

### 4.2 Natural Polyphenols

Naturally derived polyphenols have been extensively investigated for their antioxidant, anti‐inflammatory, antitumour and antibacterial properties (Yu et al. 2025; Zhong et al. 2022; Scarabelli et al. 2009). These properties are largely attributable to their chemical structure, which is typically characterized by multiple hydroxyl groups and aromatic rings that enable redox activity and molecular interactions with proteins, enzymes and other cellular targets. Such interactions underline many of their therapeutic effects and contribute to their ability to modulate signalling pathways and oxidative stress responses (Bolaños‐Cardet et al. 2026; Shen et al. 2022). However, their chemical structure is also responsible for their low solubility and polarity, which hinders their absorption through biological barriers and reduces their bioavailability (Yu et al. 2025). These characteristics, together with rapid metabolism, limit the clinical applicability of natural polyphenols.

In recent years, BMEVs have emerged as effective carriers for polyphenols. Multiple studies have shown that encapsulation into BMEVs can enhance the solubility, stability and bioavailability of curcumin, one of the most widely investigated polyphenolic compounds. Compared with the free molecule, BMEV‐mediated delivery improves gastrointestinal transport and systemic absorption, indicating that vesicular encapsulation protects the compound during transit through harsh physiological environments (Schifano et al. 2025; Wu et al. 2025; Carobolante et al. 2020). In addition to improving pharmacokinetics, BMEV loading can preserve or even enhance the biological activity of polyphenols. For instance, curcumin delivered via BMEVs retained its antioxidant and anti‐inflammatory properties and showed improved therapeutic efficacy in a sodium iodate‐induced model of retinal pigment epithelial cell damage (Wu et al. 2025). In vivo evidence further supports these findings revealing that administration of curcumin‐loaded BMEVs in worm models resulted in rapid distribution across multiple tissues and amplified biological effects compared with free curcumin (Schifano et al. 2025).

Further confirmation of the protective and delivery capabilities of BMEVs comes from studies demonstrating rapid tissue accumulation of vesicle‐encapsulated polyphenols. Curcumin and resveratrol were detected in rat mammary glands within minutes after administration when delivered through BMEVs, whereas free compounds were undetectable (González‐Sarrías et al. 2022). These results indicate that vesicular encapsulation can shield polyphenols from rapid metabolic degradation and facilitate efficient systemic distribution. Consistently, the same investigation reported enhanced antiproliferative activity of BMEV‐loaded curcumin and resveratrol in breast cancer cell lines compared with their non‐encapsulated counterparts.

Additional evidence comes from Lou et al., who used BMEVs to deliver epicatechin gallate, resulting in improved neuroprotective effects in a rotenone‐induced Parkinson's disease model compared with the free compound (Luo et al. 2021).

### 4.3 Nucleic Acids

MicroRNAs (miRNAs) and small interfering RNAs (siRNAs) are key effectors of RNA interference‐based therapies (Traber and Yu 2023; Kara et al. 2022). miRNAs post‐transcriptionally regulate gene expression, whereas siRNAs silence specific genes by binding complementary mRNA sequences. These approaches enable the selective targeting of disease‐relevant genes (Yang et al. 2023; Zhao et al. 2019; Dzau and Hodgkinson 2024). However, RNA‐based therapies face low cellular uptake due to the negative charge of nucleic acids and present low stability because of the presence of RNases within cells (Kara et al. 2022). To overcome these drawbacks, BMEVs have emerged as a promising platform for nucleic acid delivery. Several studies have demonstrated the feasibility of loading BMEVs with different types of RNAs through electroporation or lipofectamine treatment. BMEVs can functionally deliver and protect the loaded nucleic acids from RNSase. This protective effect is primarily attributed to vesicular encapsulation of RNA within the lipid bilayer, whereas nucleic acids potentially associated with the vesicle surface may remain more susceptible to enzymatic degradation (Del Pozo‐Acebo et al. 2021; Roerig et al. 2022). Yan et al. observed that miR‐31‐5p‐loaded BMEVs delivered functional miRNA to human endothelial cells HUVECs, improving their proliferation, migration and tube formation compared with free miRNA in vitro and accelerated wound healing in a diabetic mouse model in vivo (Yan et al. 2022). Kim et al., developed a shock wave‐based method to load KRAS^G12C^‐targeting siRNA into BMEVs This approach reduced KRAS^G12C^ expression and inhibited tumour cell growth in a dose‐dependent manner, both in vitro and in vivo (Kim et al. 2024).

Collectively, these findings underscore the considerable potential of BMEVs as versatile delivery platforms for small molecules, natural polyphenols and nucleic acid therapeutics, owing to their ability to enhance bioavailability, protect cargo from degradation, and enable functional delivery. Nevertheless, further studies are required to clarify loading mechanisms, optimize efficiency and comprehensively evaluate pharmacodynamics, safety and translational applicability across models.

## 5 Patents Landscape Overview

**FIGURE 4:**
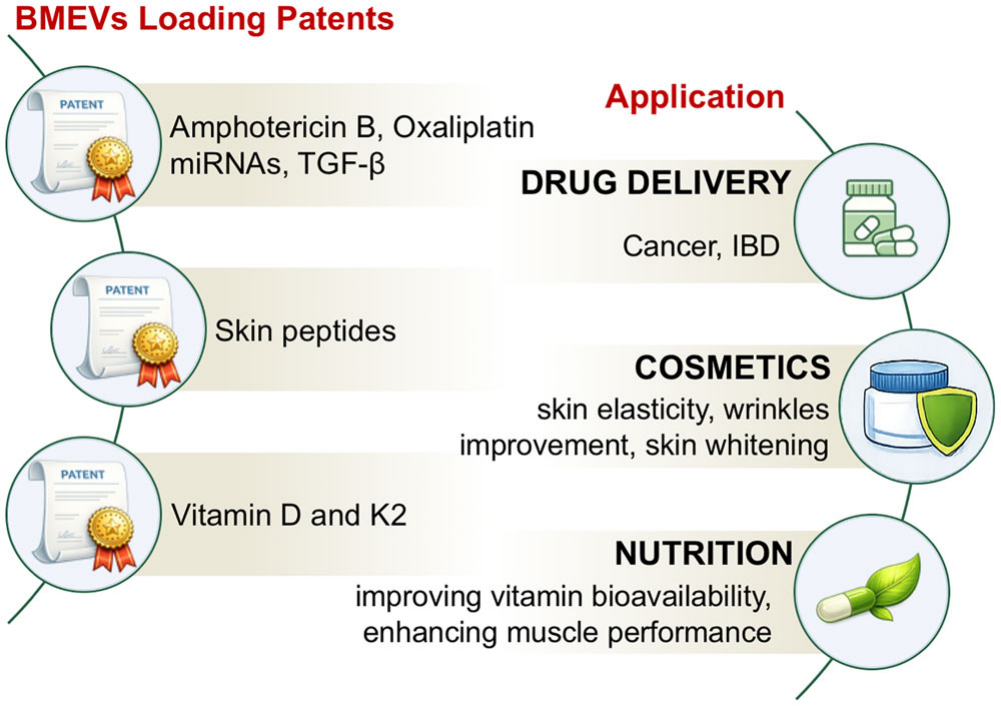
Examples of patented formulations containing BMEVs in the therapeutic, cosmetics and nutraceutical fields. In therapeutic applications BMEVs are used as nanocarriers to deliver drugs such as Amphotericin B and Oxaliplatin or molecules like miRNAs and TGF‐β. In the cosmetics field they are applied alone or loaded with skin peptides to alleviate wrinkles and improve skin elasticity. In the nutraceutical industry, BMEVs are used to enhance vitamin bioavailability or formulated as EVs enriched powders to enhance muscle performance.

The growing scientific interest in BMEVs is reflected in a rising number of patents, encompassing both the optimization of vesicle purification methods and the development of novel formulations for applications in the cosmetic, nutraceutical and pharmaceutical sectors (Figure 4). Recent patents describe high‐purity EV production processes and therapeutic formulations loaded with antifungal drugs, microRNAs, growth factors or targeted chemotherapeutics for conditions such as infections, inflammatory diseases and cancer. Additional filings report cosmetic preparations aimed at improving skin elasticity, reducing wrinkles or treating pigmentation disorders, as well as nutraceutical formulations designed to enhance vitamin bioavailability or support muscle performance (https://worldwide.espacenet.com) (Table 3).

**TABLE 3:** Patent summary on bovine milk‐derived extracellular vesicles.

Application field	Patent	Description	Cargo/Active component	Intended use
Isolation/Manufacturing Methods	CH719081A2	Scalable isolation of EVs	Amphotericin B	Oral anti‐infective therapy
Isolation/Manufacturing Methods	CN118961854A	Extraction and identification of EVs	N/A	Isolation and identification
Isolation/Manufacturing Methods	CN121046293A	Improved casein removal	N/A	High‐yield isolation
Drug Delivery Platforms	US10420723B2	Milk EV drug carriers	Chemotherapeutics	Drug delivery
Drug Delivery Platforms	US20230157955A1	Oral delivery vesicles	Bioactive compounds	Oral delivery
Drug Delivery Platforms	US20250360169A1	Exosome delivery systems	miRNA	Delivery to mammalian cells
Drug Delivery Platforms	EP3620519A1	Oral delivery of nucleic acids	ASO	Oral delivery to tissues
Targeted/Engineered EVs	KR20230105122A	GE11‐targeted EVs	GE11‐oxaliplatin	Targeted cancer therapy
Targeted/Engineered EVs	CN117414434	CRISPR EV carriers	CRISPR/Cas9 ± drugs	Targeted gene therapy
Targeted/Engineered EVs	CN119055683	Osteoblast‐targeting EVs	Bone peptide	Bone regeneration
Intrinsic Therapeutic EVs	CA3139087A1	EVs for IBD	Native EVs	IBD treatment
Intrinsic Therapeutic EVs	WO2024181919A1	Gut barrier EVs	Native EVs	Gut/liver therapy
Intrinsic Therapeutic EVs	WO2022215925A1	Osteogenesis EVs	Native EVs	Bone growth
Intrinsic Therapeutic EVs	EP3969019	EVs for IBD	Native EVs	IBD treatment
Intrinsic Therapeutic EVs	US20230338425A1	Immune modulation EVs	Native EVs	Immune enhancement
Immunotherapy	US8932855B2	Immune exosomes	Cancer antigens	Cancer immunotherapy
Hybrid Systems	CN117467656A	EV‐coated probiotics	Probiotics	Oral probiotic delivery
Cosmetic Applications	WO2020018926A1	Skin EV formulations	Skin peptides	Skin treatment
Cosmetic Applications	US2023039133A1	Skin EV compositions	Native EVs	Anti‐aging
Cosmetic Applications	WO2020218846A1	Skin whitening EVs	Native EVs	Skin whitening
Cosmetic Applications	US20210169928A1	MSC EV topical therapy	MSC EVs	Topical regenerative therapy
Nutraceutical Applications	WO2024249615A1	Vitamin‐loaded EVs	Vitamin D	Bioavailability
Nutraceutical Applications	WO2024015441A1	Vitamin‐loaded EVs	Vitamin K2	Bioavailability
Nutraceutical Applications	CA3203431	EV supplements	Native EVs	Muscle performance

*Source*: Google patents, patentscope.wipo and Espacenet‐patent search.

Taken together, the patent landscape highlights the versatility and commercial relevance of BMEVs. The diversity of patented formulations and cargo reflects both the technological progress in this field and the growing interest in translating milk‐derived vesicles into commercially available products.

Despite these advances, there are challenges that must be addressed to support further development and clinical translation. These include the need for standardized and reproducible production, characterization protocols, and a more comprehensive understanding of biodistribution and cargo release. Addressing these knowledge gaps is essential to fully understand the potential of BMEVs and to guide their development in future applications.

## 6 Critical Aspects and Future Perspectives

**FIGURE 5:**
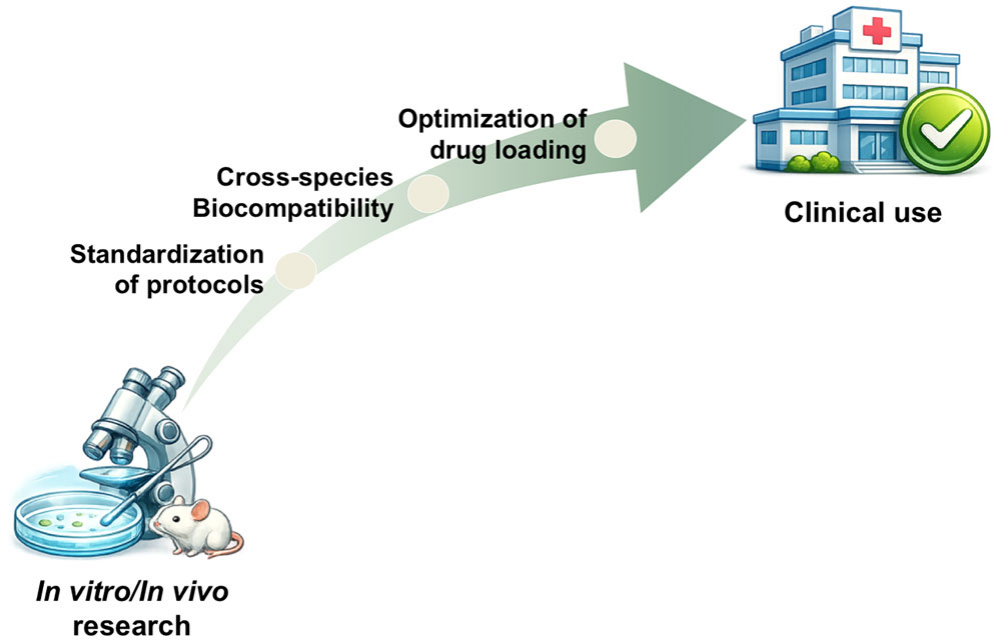
Current barriers limiting the clinical translation of BMEVs. Despite significant recent advances, further investigations are still required to support their safe and effective clinical application. Key challenges include the standardization of isolation and purification protocols, the assessment of cross‐species biocompatibility, and the optimization of cargo loading strategies.

As highlighted in this review, BMEVs represent a promising platform for drug delivery, but several critical aspects need to be elucidated (Figure 5). Standardization of isolation and purification methods is a primary hurdle, as yield and quality vary depending on protocols, which can influence purity, safety and reproducibility (Mecocci, Ottaviani, et al. 2022; Kankaanpää et al. 2024). Moreover, factors such as cow health, lactation stage and environmental conditions can influence the complex milk composition, further complicating batch‐to‐batch reproducibility (Wang et al. 2023; Rahman et al. 2021).

Another key requirement for clinical translation is the development of rapid and scalable production strategies. Currently, differential ultracentrifugation combined with size‐exclusion chromatography remains the most widely used approach in a research setting, although it is difficult to scale. Tangential flow filtration has emerged as a promising alternative; however, further research is needed to ensure high purity and to establish good manufacturing practices protocols for BMEVs isolation (Giancaterino and Boi 2023; del Saz‐Lara et al. 2025).

Toxicity and biocompatibility of BMEVs represent additional critical considerations. Although in vivo studies indicated that BMEVs are generally well tolerated, additional research is required to evaluate long‐term exposure and potential cross‐species immune responses (Somiya et al. 2018; Munagala et al. 2016). Finally, improving cargo loading efficiency while preserving vesicle integrity represents an important technical challenge for their application as drug delivery systems (Marsh et al. 2025; Jiang et al. 2024).

In addition to these technical challenges, translational progress is currently limited by the absence of clinical studies involving BMEVs and by the lack of a dedicated regulatory framework for EV‑based therapeutics

To date, no clinical trials have been conducted using BMEVs as therapeutic agents or drug delivery vehicles, highlighting that they remain at a preclinical stage of development. Early clinical investigations involving milk‑derived EVs have so far been limited to human milk EVs, such as the administration of donor human milk EVs in preterm infants. While these studies do not directly assess BMEVs, they demonstrate that milk‑derived EVs can progress toward clinical evaluation and provide a preliminary translational framework for the field.

From a regulatory perspective, major agencies such as the US Food and Drug Administration (FDA) have not yet established a dedicated category for EV‑based therapeutics. Instead, EVs are typically regulated under existing frameworks applied to biologics or, depending on source and intended use, combination products. Regulatory evaluation requires compliance with good manufacturing practices (GMP), detailed characterization of vesicle identity, purity and potency, and rigorous safety testing. For milk‑derived EVs, additional considerations include batch‑to‑batch variability linked to biological source, cross‐species immunogenicity, and the need for standardized methods ensuring reproducibility. The absence of a clear regulatory pathway represents a major barrier to the clinical progression of BMEVs, and future work will need to address these aspects to enable their translation into therapeutic applications.

To advance the field, future efforts should therefore prioritize (i) the development of standardized, reproducible and scalable isolation and purification workflows; (ii) systematic multi‐site validation of BMEV preparations to ensure robustness across laboratories; (iii) harmonized protocols for safety, bio‐distribution and pharmacokinetic assessment and (iv) establishment of GMP‐compliant production pipelines suitable for clinical‐grade material.

## 7 Conclusions

While the intrinsic properties of BMEVs position them as a competitive alternative to cell‐derived vesicles, their clinical translation hinges on addressing several unresolved bottlenecks. Future research must shift from proof‐of‐concept studies toward the standardization of isolation protocols, as current variability across procedures limits the reproducibility of therapeutic outcomes.

Furthermore, a deeper mechanistic understanding of their in vivo biological behaviour and long‐term pharmacokinetics remains essential. Optimizing cargo loading efficiency without compromising the structural integrity of the vesicles is another critical hurdle that requires innovative engineering approaches. Addressing these technical and regulatory gaps will be pivotal to transforming BMEVs from a promising laboratory platform into a reliable, large‐scale delivery system for biomedical, cosmetic and nutraceutical applications.

## Author Contributions

**Martina Brattini**: writing – original draft, conceptualization. **Michelle D'urso**: writing – original draft. **Sofia Mariotto**: conceptualization, writing – review and editing, funding acquisition, supervision. **Elena Butturini**: writing – review and editing, conceptualization, supervision.

## Funding

This work was supported by funds from Ministero dell'Università e della Ricerca (FUR2024SM and FUR2024EB) and DM118/2023, PNRR missione 4.

## Conflicts of Interest

The authors declare no conflicts of interest.

## Data Availability

Data sharing not applicable to this article as no datasets were generated or analysed during the current study.
